# Effect of ultrasound technique to improve quality of Iranian industrial honey by controlling crystallization process

**DOI:** 10.1002/fsn3.3974

**Published:** 2024-01-22

**Authors:** Safa Rabbani, Seyed Amir Ali Anvar, Sara Allahyaribeik, Behrooz Jannat, Hamed Ahari

**Affiliations:** ^1^ Department of Food Hygiene, Science and Research Branch Islamic Azad University Tehran Iran; ^2^ Department of Energy and Industry, Faculty of Natural Resources and Environment, Science and Research Branch Islamic Azad University Tehran Iran; ^3^ Food and Drug Deputy Ministry of Health, and Medical Education Tehran Iran; ^4^ Department of Food Science and Technology, Science and Research Branch Islamic Azad University Tehran Iran

**Keywords:** antimicrobial activity, antioxidant activity content, crystallization, Iranian industrial honey

## Abstract

This experiment aimed to assess the effects of ultrasound techniques on the quality of Iranian industrial honey. Honey samples were subjected to ultrasound waves at different frequencies and various parameters. The results showed that both ultrasound treatments (30 or 42 kHz) changed the physical, biochemical, antioxidant, and antibacterial characteristics of honey. Ultrasound treatments at 20 or 45°C for 1, 5, or 10 min reduced moisture, acidity, sugars, ABTS levels, 5‐hydroxymethylfurfural content, clostridium, aerobic mesophilic bacteria count, and osmophile count while increasing diastase, phenol, and proline levels. Ultrasound treatment of honey samples at 30 and 42 kHz and different temperatures for varying durations led to a decrease in acidity after 90 and 180 days. Treating honey samples with 42 kHz ultrasound at 45°C for 10 min led to a significant reduction in the amount of reducing sugar. Ultrasonication at different frequencies and temperatures led to higher levels of phenol, ABTS, and proline production, along with a considerable decrease in the total count of aerobic mesophilic bacteria. Our study unveils the potential of ultrasonication to enhance honey quality through multifaceted improvements. Treatment significantly augmented phenolic content and antioxidant capacity, opening avenues for novel honey preservation and quality enhancement strategies. Additionally, ultrasonication effectively controlled honey crystallization while simultaneously improving biochemical, antioxidant, and antibacterial properties. This demonstrates its potential as a comprehensive strategy for honey quality improvement.

## Graphical abstract

This study evaluated how ultrasound waves at different frequencies and temperatures affected the quality of Iranian industrial honey. Various parameters were measured on days 1, 30, 90, and 180 after ultrasound treatments. The results showed that ultrasound treatments changed the physical, biochemical, antioxidant, and antibacterial characteristics of honey. Ultrasound techniques can improve honey quality by controlling its crystallization and avoiding the drawbacks of other processing methods.

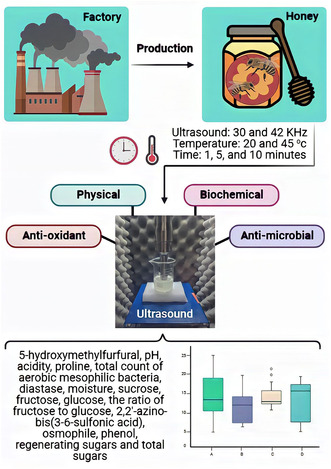

## 1 INTRODUCTION

Extensive research has focused on enhancing the nutritional value, safety, and taste of food in recent years (Ahari, Anvar, Ataee, et al., 2021). Honey is a nutritious food with significant positive effects on the human body (Al‐Farsi et al., 2018; Chuttong et al., 2016; Dabbagh Moghaddam et al., 2021; Moghaddam et al., 2020; Özcan & Ölmez, 2014; Sakač et al., 2019). It is a supersaturated glucose solution that naturally crystallizes at room temperature (Aurongzeb & Azim, 2011). Honey's natural tendency to crystallize poses a major challenge for the food industry, as it thickens and clings to machinery, disrupting processing and handling (Gośliński et al., 2023; Haji et al., 2023).

In fact, in the food industry, several parameters are considered to estimate the characteristics of honey (Moumeh et al., 2020). One of these parameters is the amount of 5‐hydroxymethylfurfural (HMF) created as a carcinogenic compound after thermal processing. The high concentration of HMF also darkens honey's color and reduces honey's quality (Önür et al., 2018). Another parameter is diastase, which reports α‐amylase activity and is shown as milliliters of 1% starch hydrolyzed by the enzyme in 1 g of honey in 1 h. Subsequently, the remaining starch content shows a blue color complex in contact with an iodine solution. The characteristics of honey are greatly affected by its storage conditions, including temperature, pH, and sugar content. When stored in nonstandard conditions, it can lead to a higher concentration of various furfural derivatives formed due to the degradation of reducing sugars (Pauliuc et al., 2020). In addition, a survey of honey consumers reported that most people prefer to use liquid honey rather than crystallized honey in their food routines (Żak, 2017). Heat processing remains the standard method for controlling honey crystallization and preventing product issues (Grabowski & Klein, 2017; Subramanian et al., 2007).

Though widely employed in honey processing, thermal treatment's appeal is tarnished by its well‐documented detrimental impact on food's nutritional and functional aspects. This stems from the intricate interplay between different food components, creating a vulnerable and complex network susceptible to degradation under heat (Faraz et al., 2023). As a result, new methods have been investigated in many food industries and the scientific community to control and prevent these adverse effects after using the thermal method in honey (Scripcă & Amariei, 2021). One of these strategies was the use of ultrasound, which can reduce honey viscosity and liquefaction while minimizing the negative consequences of thermal processing (Chen et al., 2021). Compared to conventional heating methods, ultrasonic decrystallization offers a low‐temperature approach for honey liquefaction, thereby mitigating the thermal degradation of bioactive components such as enzymes and vitamins (Anjaly et al., 2022; Pandiselvam et al., 2022; Taha et al., 2022). Cavitation phenomena induced by ultrasonic waves facilitate rapid and volumetric heating, leading to significantly faster decrystallization kinetics (Dżugan et al., 2021; Sidor et al., 2021). Traditional heating methods for honey suffer from uneven treatment due to thermal gradients. Consequently, some regions overcook, compromising quality, while others remain underheated, impacting crystal dissolution and microbial control. Ultrasonic processing, operating in continuous flow, ensures uniform sonication throughout the honey, enabling precise control over liquefaction, decrystallization, and stabilization (Owayss et al., 2020; Pauliuc et al., 2020).

Among widely traded food products, honey, long considered a symbol of natural purity, unfortunately shares the dubious distinction of being heavily adulterated alongside milk and olive oil. This concerning reality has spurred global trade and food regulatory agencies to prioritize honey's quality assurance and safety. Emerging technologies are transforming the honey industry by improving the quality, safety, and traceability of bee products. Advanced food processing and omics technologies are being used to enhance nutritional value, reduce the risk of foodborne illness, and authenticate honey. By leveraging these technologies, beekeepers and honey producers can provide consumers with high‐quality honey that is safe to consume (Brar et al., 2023; Scepankova et al., 2021).

The scarcity of research motivates this investigation into the heretofore uncharacterized effects of ultrasound on honey's quality and biochemical profile. Ultrasound has the potential to improve the quality of honey without compromising its nutritional value or introducing harmful chemicals. This research assessed the effect of ultrasound techniques on Iranian industrial honey by controlling the crystallization process.

## 2 MATERIALS AND METHODS

Fresh Iranian honey samples (500 g each) from Tehran supermarkets were subjected to varying ultrasound treatments: frequencies (30 and 42 kHz), temperatures (20 and 45°C), and durations (1, 5, and 10 min). These were conducted in an ultrasonic bath with a thermostat and followed by storage in light‐protected containers at 18–20°C. The physicochemical and microbiological properties of both treated and control samples were analyzed at regular intervals (1, 30, 90, and 180 days) for parameters like HMF (a marker of heat degradation), pH, acidity, proline, diastase (a freshness indicator), moisture, sugars (sucrose, fructose, glucose), fructose‐to‐glucose ratio, antioxidants (ABTS and phenol), and reducing/total sugars. This multi‐parameter analysis aims to identify optimal ultrasound settings for effective honey decrystallization while preserving its quality and safety.

### 2.1 Moisture content

Honey's water activity, closely tied to its moisture content, governs its microbial stability. By controlling water availability, it effectively inhibits undesirable microbial growth during storage. High‐quality honey typically contains less than 20% water, with the ideal moisture content ranging between 16% and 18%. An Abbe refractometer (RE40; Mettler Toledo) calibrated with certified standards was employed to measure the refractive index of honey samples, facilitating the subsequent calculation of moisture content.

### 2.2 pH and acidity rates

Honey pH, dictated by the floral source of nectar, typically ranges from 3.4 to 6.1 (average 3.9). This acidic range, optimal between 3.4 and 4.5, inhibits most bacterial growth. Ten grams of each honey sample were dissolved in distilled water for pH and acidity assessments. pH was measured at 20°C, while acidity was determined by titrating the solution to pH 8.3 with 0.1 N NaOH, using phenolphthalein as an indicator.

### 2.3 Glucose, fructose, and fructose–glucose ratio

Honey's primary components are sugars, with glucose and fructose forming up to 83% of its weight. An ideal 1:1 ratio of fructose to glucose contributes to honey's quality. To measure sugar levels, a dissolved honey sample in 25 mL of distilled water was mixed with 0.1% iodine solution and 20 mL of 0.5 M NaOH. After incubating in the dark for 15 min, the sugar content was analyzed utilizing HPLC 1260 (Agilent Scientific Instruments, USA) following established methods (Scripcă & Amariei, 2021).

### 2.4 Total and reducing sugars

Natural honey, primarily composed of glucose and fructose with low levels of sucrose and maltose, must contain more than 60% reducing sugars (calculated as invert sugar) according to honey standards. Reducing sugars were quantified using the Lane‐Eynon technique with Fehling's solution and titration as described previously (Afshari et al., 2022).

### 2.5 5‐Hydroxymethylfurfural

Honey and processed foods harbor a hidden compound called HMF, born from the heat‐driven Maillard reaction between their reducing sugars and acidity. The presence of HMF in honey indicates its quality and freshness. The levels of HMF were determined using the Winkler spectrophotometric method (Scripcă & Amariei, 2021) with a Shimadzu UV‐3600i Plus (Japan) at a wavelength of 550 nm. HMF exhibits a proportional relationship with the red complex formed during the barbituric acid‐para‐toluidine assay, allowing for quantitative HMF determination.

### 2.6 Diastase activity

Diastase activity, a vital enzyme in honey, serves as a key indicator of freshness and heat exposure. It naturally degrades over time, especially with heat, and is influenced by factors like botanical origin, geography, and harvest time. In this study, we employed a standard protocol established by Horwitz (1975) to measure diastase activity in industrial honey samples. This method involved dissolving 10 g of honey in distilled water, bringing the volume to 100 mL, and mixing it with a starch solution. The mixture was then incubated at a constant temperature of 48°C in a water bath. The complete hydrolysis of 1 g of honey was visually determined by observing the formation of a blue color, and the required volume of starch solution was calculated accordingly.

### 2.7 Proline activity

Proline is an amino acid that bees secrete through their hypopharyngeal glands and is naturally present in honey. The proline content in honey varies depending on the processing time of nectar by bees and the floral type of honey. Low proline values in honey may indicate possible adulteration. To begin the experiment, three test tubes were prepared, each containing 0.5 mL of industrial honey solution, 0.5 mL of proline, and 0.5 mL of distilled water. Subsequently, 1 mL of ninhydrin formic acid was added to each tube, which was then centrifuged for 15 min. The tubes were submerged in a preheated water bath maintained at 70°C for 5 min. Afterward, 5 mL of 2‐propanol was introduced to each tube and left at 25°C. Finally, the absorption of the tubes was evaluated at a wavelength of 520–500 nm.

### 2.8 Total phenolic content

The presence of phenolic compounds in honey is intrinsically linked to its antioxidant activity and sensory characteristics. Notably, these constituents function as valuable biomarkers for both floral and geographical origin determination, with their total content exhibiting pronounced variation contingent upon the honey's specific floral source and geographical context. The phenol content of each industrial honey sample was measured based on the recent study conducted by Horwitz (1975).

### 2.9 ABTS

ABTS is a common method used to measure the antioxidant capacity of honey samples. The ABTS content of each industrial honey sample was measured based on the protocol performed in a previous study (Cheng et al., 2019).

### 2.10 Antimicrobial properties

The complex interplay between honey's high sugar content, which promotes microbial growth, and its inhibitory components, including hydrogen peroxide and polyphenols, determines its susceptibility to specific pathogens. However, the amount of contamination of honey with pathogenic bacteria and fungi is often very small, and they only remain in an inactive form in honey. If the moisture content of honey increases to more than 18%, a series of osmophilic bacteria and sugar‐loving fungi begin to grow and multiply, leading to honey fermentation and spoilage. Most kinds of honey produced in Iran have a moisture content of less than 17%, and only honeys from humid regions and forests in the north of the country, such as citrus honey and humid honey, or unripe honeys, have more moisture, and there is a possibility of fermentation. The antimicrobial rate of each industrial honey sample was measured based on the recent study conducted by Wahab et al. (2018).

### 2.11 Statistical analysis

Experimental results are depicted as mean ± standard deviation, and graphs were generated using GraphPad Prism 9. Statistical comparisons were performed using a two‐way ANOVA with Tukey's post‐hoc test, with statistical significance considered at *p* ≤ .05.

## 3 RESULTS

### 3.1 Moisture

The results of a moisture test on Iranian industrial honey samples showed that treating them with 30 and 42 kHz ultrasound (at 20°C) led to a meaningful reduction in moisture levels in comparison to the control (*p* < .0001) on days 1, 30, 90, and 180. The sample managed with 42 kHz ultrasound presents lower moisture levels in comparison to the one treated with 30 kHz ultrasound (at the same temperature and time) on days 1, 30, 90, and 180 (*p* < .05 and *p* < .01, respectively). When the temperature was increased to 45°C for 10 min and samples were treated with 30 and 42 kHz ultrasound, moisture content decreased in both groups in comparison to the control group on days 1, 30, 90, and 180 (*p* < .001). It can be concluded that treating Iranian industrial honey samples with 30 and 42 kHz ultrasound at 20°C (for 5 and 10 min) or 45°C (for 10 min) can effectively reduce moisture content.

### 3.2 pH

After being subjected to ultrasound treatments at 20°C, industrial honey samples from Iran presented a considerable increase in pH rates in comparison to the control group on day 180. The increase was meaningful (*p* < .001 and *p* < .0001) for both frequencies. In comparison to the 30 kHz ultrasound treatment, the 42 kHz ultrasound treatment resulted in a higher pH rate on day 180 for 1 and 10 min (*p* < .01 and *p* < .001), respectively. Similarly, treating honey samples at a temperature of 45°C for 1, 5, and 10 min with 30 and 42 kHz ultrasound resulted in a significant increase in pH rate compared to the control group on day 180 (*p* < .01 and *p* < .0001); (*p* < .001 and *p* < .0001); (*p* < .01 and *p* < .001), respectively. On day 180, the 42 kHz ultrasound treatment resulted in a higher pH rate compared to the 30 kHz ultrasound treatment at a temperature of 45°C for 1, 5, and 10 min (*p* < .01, *p* < .01, and *p* < .05), respectively. Therefore, it can be concluded that treating honey samples with 30 and 42 kHz ultrasound at temperatures of 20 and 45°C for 1, 5, and 10 min after 180 days can increase the pH rate of the honey.

### 3.3 Acidity

**FIGURE 1:**
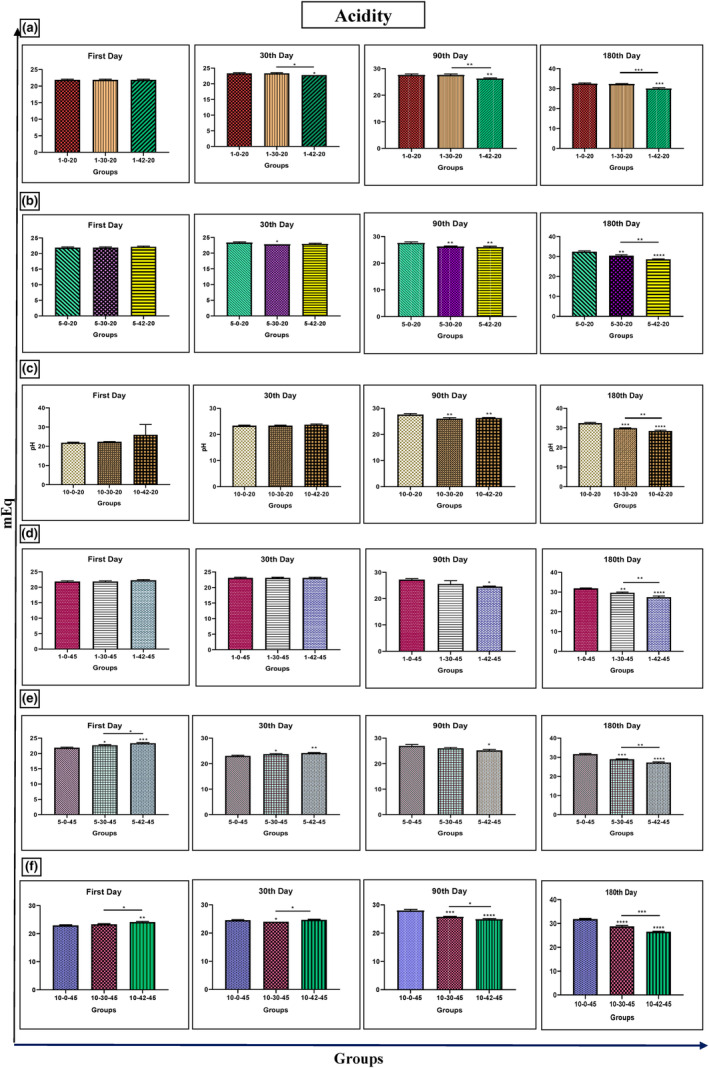
Changes in acidity in Iranian industrial honey samples after treatment with 0 (as control), 30, and 42 kHz ultrasound. The samples that were treated at 30 kHz for 1 (a), 5 (b), and 10 (c) minutes, and 20°C. The samples that were treated at 45 kHz for 1 (d), 5 (e), and 10 (f) minutes, and 45°C. Data are shown as the mean ± SD. The mean values with asterisks are significantly different (*p* ≤ .05) (*p < .05; **p < .01; ***p < .001; ****p < .0001).

Ultrasound treatment significantly reduced honey acidity, regardless of frequency (30 or 42 kHz) or temperature (20 or 45°C). Both short (1 min) and longer (5 and 10 min) treatments were effective, with the greatest reductions observed after longer storage times (90 and 180 days) (Figure 1).

### 3.4 Glucose, fructose, rate of fructose to glucose, sucrose, and total sugar

Honey samples treated with 30 and 42 kHz ultrasound at a temperature of 20 and 45°C for 1, 5, and 10 min after 1, 30, 90, and 180 days showed no glucose, fructose, fructose to glucose, sucrose, or total sugar rate changes.

### 3.5 Reducing sugar

Sonication at 42 kHz and 45°C for 10 min proved a potent tool for reducing sugar levels in Iranian industrial honey. Samples subjected to this treatment maintained significantly lower (*p* < .01) levels of reducing sugars compared to the untreated control group across all studied storage intervals (days 1, 30, 90, and 180). Moreover, the sample treated with 42 kHz ultrasound had a lower reducing sugar rate (*p* < .01) than the one treated with 30 kHz at the same temperature and duration on the same days.

### 3.6 5‐Hydroxymethylfurfural

**FIGURE 2:**
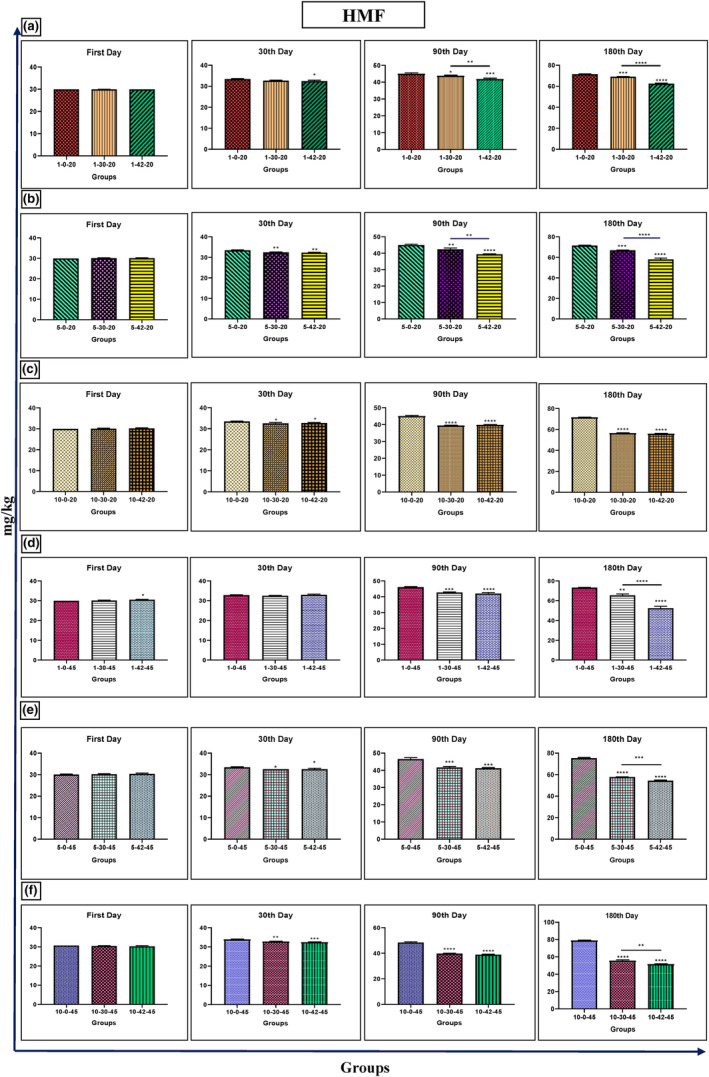
Changes of HMF in Iranian industrial honey samples after treatment with 0 (as control), 30, and 42 kHz ultrasound. The samples that were treated at 30 kHz for 1 (a), 5 (b), and 10 (c) minutes, and 20°C. The samples that were treated at 45 kHz for 1 (d), 5 (e), and 10 (f) minutes, and 45°C. Data are shown as the mean ± SD. The mean values with asterisks are significantly different (*p* ≤ .05) (*p < .05; **p < .01; ***p < .001; ****p < .0001).

Ultrasound frequencies were applied at different temperatures, and the levels of HMF were significantly reduced after 90 and 180 days (Figure 2). The treatment groups subjected to 45°C for 1, 5, and 10 min also showed a decrease in HMF. This trend was observed on the 90th and 180th days. Additionally, a significant reduction in HMF was observed after 1, 5, and 10 days of exposure to 42 kHz ultrasound at 45°C (Figure 2). The levels of HMF were significantly reduced after 90 and 180 days of exposure to 42 kHz ultrasound at 20°C for 1, 5, and 10 days (41.79 and 62.50 mg/kg); (42.42 and 39.42 mg/kg); (66.87 and 58.01 mg/kg) compared to control. Furthermore, a decrease in HMF was observed in these treatment groups (65.37, 54.39, and 51.65) after exposure to 42 kHz ultrasound at 45°C for 1, 5, and 10 min after 180 days (Figure 2).

### 3.7 Diastasis

**FIGURE 3:**
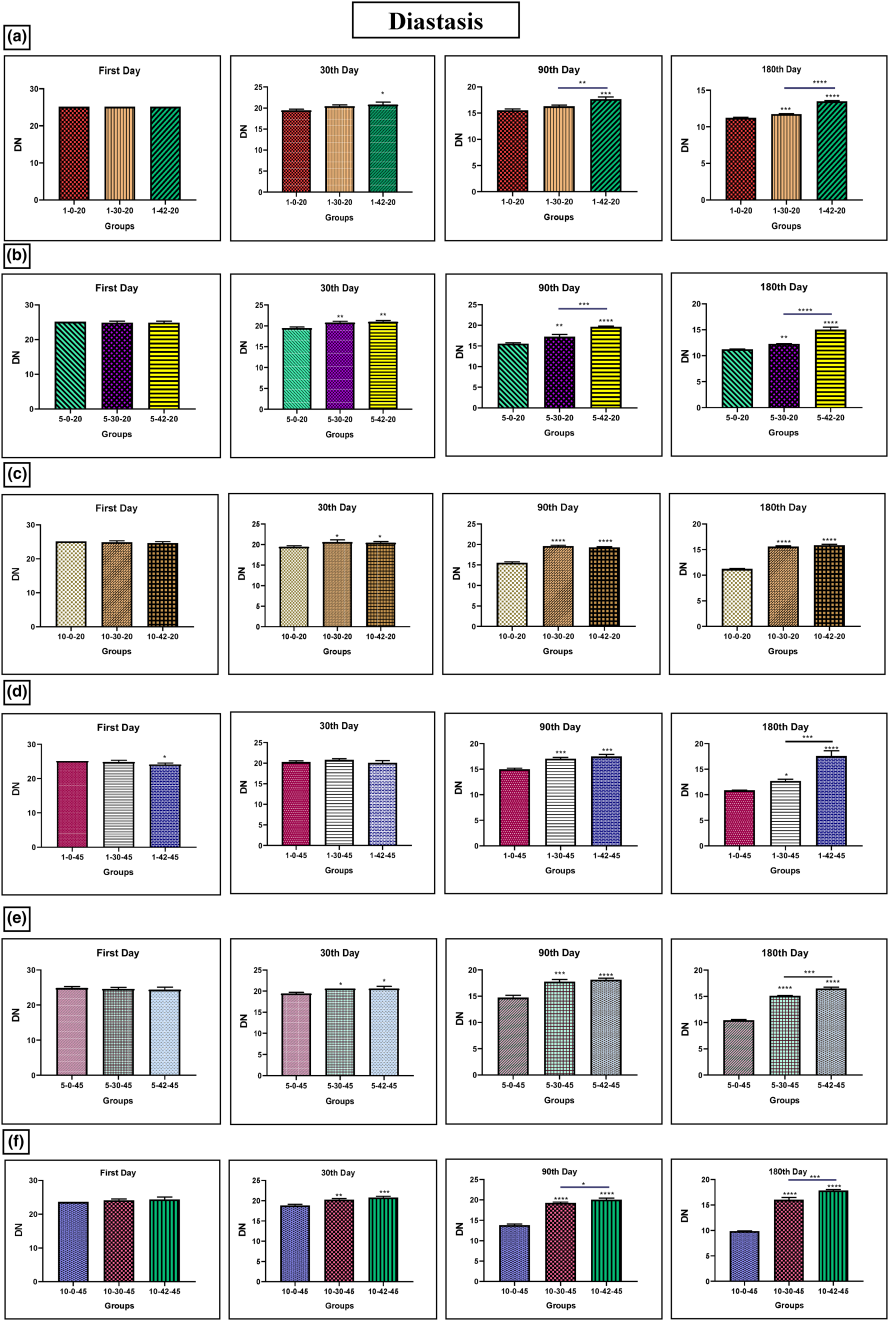
Changes of diastasis in Iranian industrial honey samples after treatment with 0 (as control), 30, and 42 kHz ultrasound. The samples that were treated at 30 kHz for 1 (a), 5 (b), and 10 (c) minutes, and 20°C. The samples that were treated at 45 kHz for 1 (d), 5 (e), and 10 (f) minutes, and 45°C. Data are shown as the mean ± SD. The mean values with asterisks are significantly different (*p* ≤ .05) (*p < .05; **p < .01; ***p < .001; ****p < .0001).

Honey samples treated with 30 and 42 kHz ultrasound at 20°C for 1, 5, and 10 min showed an increase in diastasis rate on days 90 and 180 compared to the control group (Figure 3a–c). The sample treated with 42 kHz ultrasound compared to 30 kHz ultrasound at the same temperature for 1 and 5 min also showed an increase in diastasis rate on days 90 and 180 (Figure 3a–c). Furthermore, the samples treated with both frequencies at the temperature of 45°C for the same duration showed an increase in diastasis rate on days 90 and 180. The sample treated with 42 kHz ultrasound compared to the one treated with 30 kHz ultrasound at a same temperature for the same duration showed a higher rate of diastasis on day 180 (Figure 3d–f).

### 3.8 Total phenolic content

**FIGURE 4:**
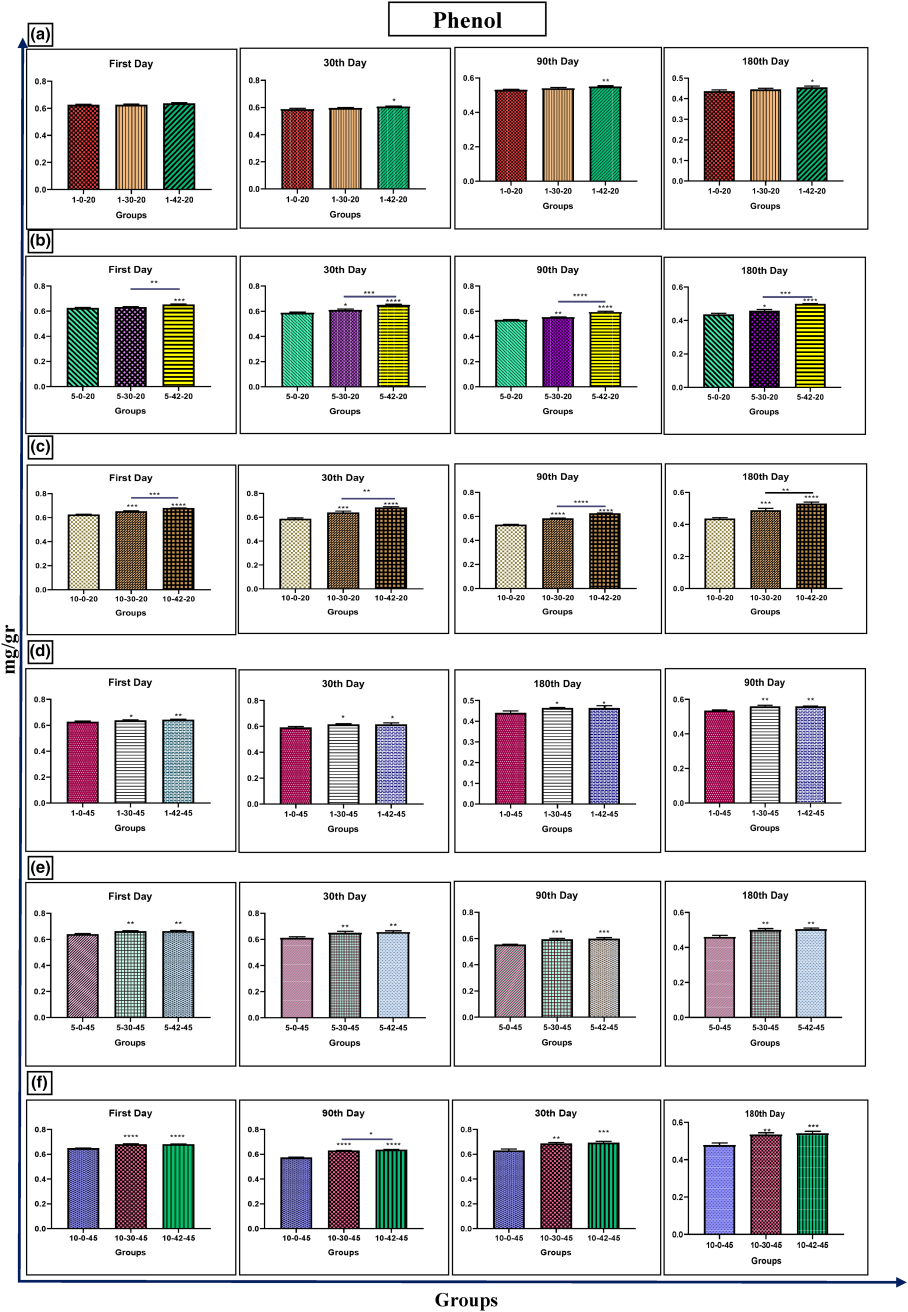
Changes of phenol in Iranian industrial honey samples after treatment with 0 (as control), 30, and 42 kHz ultrasound. The samples that were treated at 30 kHz for 1 (a), 5 (b), and 10 (c) minutes, and 20°C. The samples that were treated at 45 kHz for 1 (d), 5 (e), and 10 (f) minutes, and 45°C. Data are shown as the mean ± SD. The mean values with asterisks are significantly different (*p* ≤ .05) (**p < .01; ***p < .001; ****p < .0001).

After exposing samples to 30 and 42 kHz ultrasounds for 1 and 5 min at a temperature of 20°C on days 30, 90, and 18, there was a noticeable rise in phenol content. In addition, when samples were treated with 42 kHz ultrasound for 5 min at 20°C, the increase in phenol content was significant (*p* < .0001). Meanwhile, honey treatment at 20°C for 10 min on days 90 and 180 also significantly increased phenol content in comparison to the control group (*p* < .0001). The same rise in phenol content was observed in samples treated with 30 and 42 kHz ultrasound at 45°C for 1, 5, and 10 min (*p* < .001) (Figure 4).

### 3.9 Proline

**FIGURE 5:**
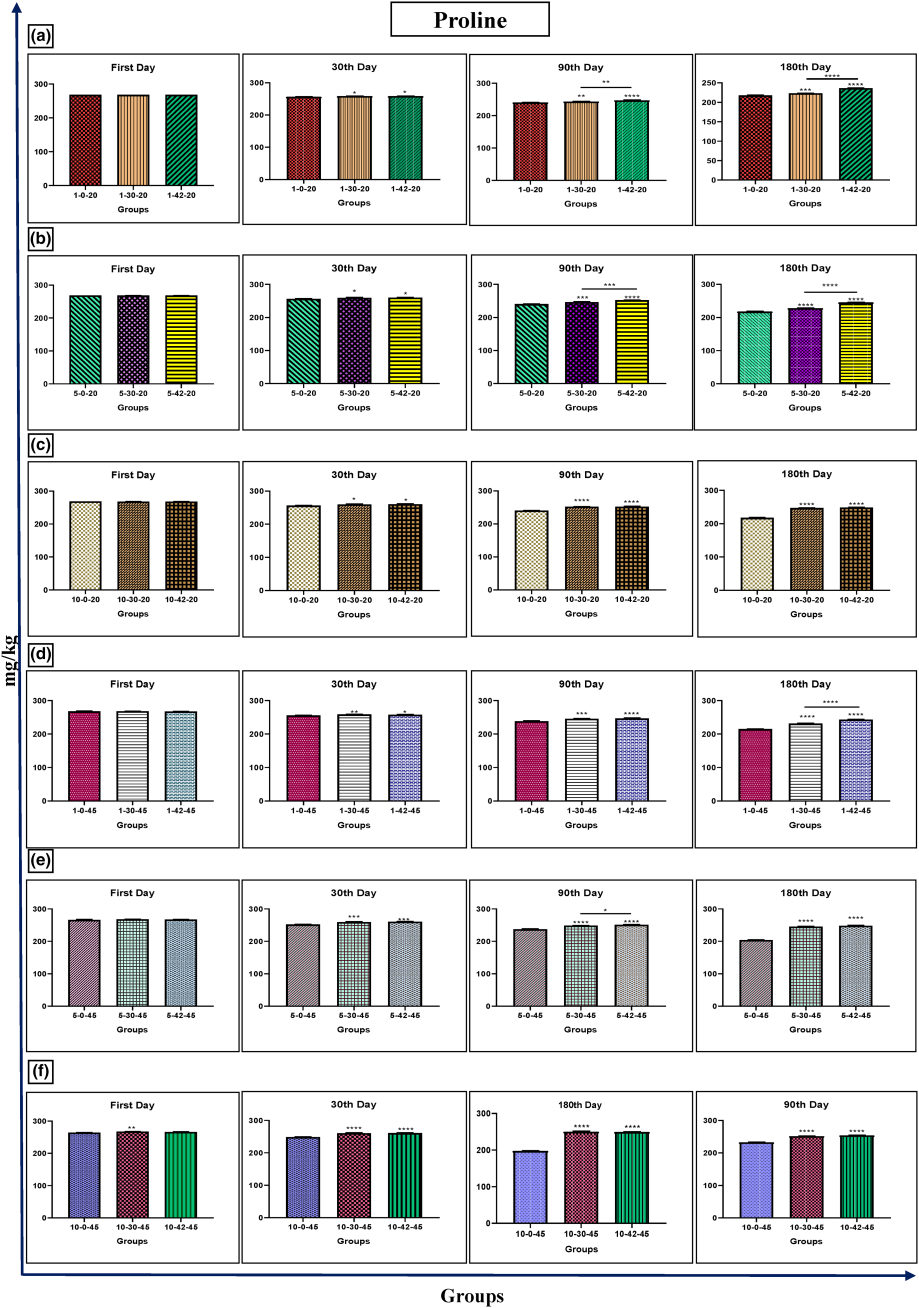
Changes of proline in Iranian industrial honey samples after treatment with 0 (as control), 30, and 42 kHz ultrasound. The samples that were treated at 30 kHz for 1 (a), 5 (b), and 10 (c) minutes, and 20°C. The samples that were treated at 45 kHz for 1 (d), 5 (e), and 10 (f) minutes, and 45°C. Data are shown as the mean ± SD. The mean values with asterisks are significantly different (*p* ≤ .05) (*p < .05; **p < .01; ***p < .001; ****p < .0001).

The honey samples' diastasis rate was treated with 30 and 42 kHz ultrasound at 20 and 45°C in comparison to the control and other study groups shown in Figure 5.

### 3.10 ABTS

Ultrasound (30 and 42 kHz) for 10 min at 20°C reduced ABTS in honey samples. On the 180th day, in these groups at 45°C, a decrease in ABTS was also noticed compared to the control.

### 3.11 Total count of aerobic mesophilic bacteria

42 kHz with a temperature of 20°C compared to the control group and 30 kHz after 1 min on the 180th day could reduce the number of aerobic mesophilic bacteria (*p* < .0001) (Table 1).

**TABLE 1:** Changes in total count of aerobic mesophilic bacteria in Iranian industrial honey samples after treatment with 0 (as control), 30, and 42 kHz ultrasound.

Tukey's multiple comparisons test, time (min)–intensity (kHz)–temperature (°C)	Treatment period (days)	Mean difference	95.00% CI of the difference	Significant	*p* Value
1–0–20 vs. 1–30–20 1–0–20 vs. 1–42–20 1–30–20 vs. 1–42–20	1	1	−5.628 to 7.628	No	.8906
		10	3.372 to 16.63	Yes	.0085
		9	2.372 to 15.63	Yes	.0139
	30	3.333	−7.030 to 13.70	No	.6108
		13.33	2.970 to 23.70	Yes	.0177
		10	−0.3631 to 20.36	No	.0571
	90	4.333	−3.720 to 12.39	No	.2972
		22.33	14.28 to 30.39	Yes	.0004
		18	9.947 to 26.05	Yes	.0012
	180	5.333	−0.4523 to 11.12	No	.0674
		23.33	17.55 to 29.12	Yes	<.0001
		18	12.21 to 23.79	Yes	.0002
5–0–20 vs. 5–30–20 5–0–20 vs. 5–42–20 5–30–20 vs. 5–42–20	1	15	10.82 to 19.18	Yes	<.0001
		19.67	15.49 to 23.84	Yes	<.0001
		4.667	0.4913 to 8.842	Yes	.0323
	30	18	11.22 to 24.78	Yes	.0005
		22.67	15.88 to 29.45	Yes	.0001
		4.667	−2.118 to 11.45	No	.1677
	90	22	14.35 to 29.65	Yes	.0003
		28	20.35 to 35.65	Yes	<.0001
		6	−1.654 to 13.65	No	.1152
	180	21.67	14.24 to 29.09	Yes	.0003
		26.67	19.24 to 34.09	Yes	<.0001
		5	−2.422 to 12.42	No	.1773
10–0‐20 vs. 10–30–20 10–0–20 vs. 10–42–20 10–30–20 vs. 10–42–20	1	16.33	12.33 to 20.34	Yes	<.0001
		22.33	18.33 to 26.34	Yes	<.0001
		6	1.995 to 10.00	Yes	.0088
	30	19.33	11.59 to 27.08	Yes	.0006
		24.67	16.92 to 32.41	Yes	.0002
		5.333	−2.411 to 13.08	No	.1672
	90	25.33	16.42 to 34.25	Yes	.0003
		29	20.08 to 37.92	Yes	.0001
		3.667	−5.250 to 12.58	No	.4641
	180	26	17.95 to 34.05	Yes	.0002
		33.33	25.28 to 41.39	Yes	<.0001
		7.333	−0.7199 to 15.39	No	.0703
1–0‐45 vs. 1–30–45 1–0–45 vs. 1–42–45 1–30–45 vs. 1–42–45	1	11	5.990 to 16.01	Yes	.0013
		13	7.990 to 18.01	Yes	.0005
		2	−3.010 to 7.010	No	.4827
	30	13	9.989 to 16.01	Yes	<.0001
		14.67	11.66 to 17.68	Yes	<.0001
		1.667	−1.344 to 4.678	No	.2806
	90	11.33	7.075 to 15.59	Yes	.0004
		14	9.742 to 18.26	Yes	.0001
		2.667	−1.591 to 6.925	No	.213
	180	11.33	4.198 to 18.47	Yes	.0067
		9	1.865 to 16.13	Yes	.0194
		−2.333	−9.468 to 4.802	No	.6016
5–0‐45 vs. 5–30–45 5–0–45 vs. 5–42–45 5–30–45 vs. 5–42–45	1	18	13.20 to 22.80	Yes	<.0001
		21.33	16.54 to 26.13	Yes	<.0001
		3.333	−1.464 to 8.130	No	.1632
	30	18.33	12.25 to 24.41	Yes	.0002
		21	14.92 to 27.08	Yes	.0001
		2.667	−3.413 to 8.746	No	.4235
	90	19	12.75 to 25.25	Yes	.0002
		22.33	16.08 to 28.58	Yes	<.0001
		3.333	−2.916 to 9.582	No	.3024
	180	16.33	9.601 to 23.07	Yes	.0007
		15.67	8.934 to 22.40	Yes	.0009
		−0.6667	−7.399 to 6.066	No	.9508
10–0‐45 vs. 10–30–45 10–0–45 vs. 10–42–45 10–30–45 vs. 10–42–45	1	19.67	11.11 to 28.22	Yes	.001
		24	15.44 to 32.56	Yes	.0003
		4.333	−4.224 to 12.89	No	.3337
	30	19	9.967 to 28.03	Yes	.0016
		22	12.97 to 31.03	Yes	.0007
		3	−6.033 to 12.03	No	.5928
	90	17.67	10.63 to 24.70	Yes	.0006
		24.33	17.30 to 31.37	Yes	.0001
		6.667	−0.3698 to 13.70	No	.0611
	180	17.33	8.072 to 26.59	Yes	.0029
		18.33	9.072 to 27.59	Yes	.0022
		1	−8.261 to 10.26	No	.9419

*Note*: Data are shown as the mean ± SD.

The mean values with asterisks are significantly different (*p* ≤ .05) (*p < .05; **p < .01; ***p < .001; ****p < .0001).

### 3.12 Mold, osmophiles, and clostridium

No meaningful changes were detected in the amount of mold, osmophilus, and clostridium in Iranian industrial honey samples.

## 4 DISCUSSION

As one of the most valuable foods, honey is highly popular among food consumers. Therefore, preserving and increasing the quality of honey is on the food industry's agenda. One of the factors that has a direct connection with the quality of honey is crystallization. To control this process, various factors must be considered and evaluated (Cheng et al., 2019). Our results showed that the ultrasound technique could have significant effects on maintaining and improving the quality of honey, which were investigated further.

Kabbani et al. conducted research on the liquefaction of rosemary honey using an ultrasound technique. The honey samples were treated with a 40 kHz ultrasonic bath at 40–60°C for 20, 40, and 60 min. The study demonstrated that ultrasound could significantly speed up the liquefaction of honey at a temperature below 50°C (Kabbani et al., 2011). Therefore, the lower temperature of 50°C that was applied in this study led to higher preservation of honey quality (decrease of moisture) compared to heat treatment. Ultrasonic cavitation induces uniform honey heating via bubble collapse, enabling rapid and efficient decrystallization. Honey moisture content dictates its microbial stability, granulation tendency, and organoleptic properties. Optimal levels below 17% limit yeast growth but conversely amplify granulation risk as moisture falls (Jiang, Bai, et al., 2021; Liu et al., 2019). Therefore, as shown in our study, ultrasonication can be an effective method for reducing the moisture content of honey by destroying microbial cells that are responsible for fermentation and water absorption.

Ultrasonication can increase the pH of honey due to its effect on the biochemistry of honey. Honey's diverse composition (sugars, enzymes, acids, etc.) reflects its floral origin, climate, and processing. Its pH (3.5–5.5) hinges on the floral source and nectar acidity (Yang et al., 2022). The quality of honey also depends on its pH and acidity (Ratiu et al., 2019). For example, acidity and pH decrease with increasing temperature in honey (65°C leads to a maximum drop in pH from 3.52 to 3.35 in raw honey) (Janghu et al., 2017). In our study, a decrease in acidity was observed in the honey samples after being treated with 30 and 42 kHz ultrasound at (20 and 45°C for 1, 5, and 10 min) after 90 and 180 days. Although honey samples treated with 30 and 42 kHz ultrasound at 20 and 45°C for 1, 5, and 10 min after 1, 30, 90, and 180 days showed no glucose, fructose, ratio of fructose to glucose, sucrose, and total sugar rate changes, a significant decrease in the amount of reducing sugar was observed after treating honey samples with 42 kHz ultrasound at 45°C for 10 min. Ultrasonication can cause the breakdown of glucose and fructose molecules in honey, leading to the formation of organic acids such as gluconic acid and formic acid. These acids can then react with other compounds in honey to form salts, which can increase the pH of honey. Additionally, ultrasonication can cause the release of enzymes such as glucose oxidase, which can catalyze the oxidation of glucose to gluconic acid and hydrogen peroxide (Scripcă & Amariei, 2021). The hydrogen peroxide can then react with other compounds in honey to form salts, which can also increase the pH of the honey (Ramly et al., 2021; Yang et al., 2022). Ultrasonication can reduce the rate of reducing sugar in honey by breaking down glucose and fructose molecules, leading to the formation of organic acids such as gluconic acid and formic acid. These acids can then react with other compounds in honey to form salts, which can reduce the rate of reducing sugar in honey (Jiang, Zhu, et al., 2021). Ultrasonication can also cause the release of enzymes such as glucose oxidase, which can catalyze the oxidation of glucose to gluconic acid and hydrogen peroxide. The hydrogen peroxide can then react with other compounds in honey to form salts, which can also reduce the rate of reducing sugar in honey (Ramly et al., 2021).

The investigation by Phawatwiangnak et al. found that color and HMF increased with the increasing strength of the ultrasonic wave, while antioxidant and diastase enzyme content decreased. However, melted honey had the highest residual antioxidant activity and diastase enzyme content, with the lowest HMF content. Melted honey also had the highest brightness level and the lowest amount of redness and yellowness (Phawatwiangnak & Intipunya, 2013). Other studies have shown that an ultrasonic process with high power in optimal conditions can maintain the quality of sunflower honey, similar to natural honey (Ismail et al., 2019; Jiang, Zhu, et al., 2021; Sidor et al., 2021). Similarly, our study found that honey samples treated with 42 kHz ultrasound at 20 and 45°C for 1, 5, and 10 min after 90 and 180 days were related to a reduction in HMF and an increase in diastasis.

Ultrasound treatment improved the quality of honey by increasing the level of phenolic compounds and antioxidant properties. The treatment also led to noticeable differences in crystal size, color, and antioxidant activity, which is in line with previous studies (Ratiu et al., 2019; Scripcă & Amariei, 2021). A study by Quintero‐Lira et al. (2017) found that a 15‐min ultrasound treatment resulted in a significant rise in phenolic acids, flavonoids, and antioxidant activity. Similarly, our study found that Iranian industrial honey samples treated with 30 and 42 kHz ultrasound at 20 and 45°C for 1, 5, and 10 min after 1, 30, 90, and 180 days were associated with an increase in phenol, ABTS, and proline rates.

The results of another study showed that ultrasound‐treated samples maintained their standard plate count parameter levels, and the yeasts and molds were undetectable (Scripcă & Amariei, 2021). The results obtained from our study showed that after treatment of Iranian industrial honey samples with 30 and 42 kHz ultrasound at 20 and 45°C for 1, 5, and 10 min and after 1, 30, 90, and 180 days, there was a considerable reduction in the total count of aerobic mesophilic bacteria levels and also no molds or clostridium detected. The total count of mesophilic bacteria is an important health indicator used to evaluate the quality and safety of honey. Mesophilic bacteria are a broad group of bacteria that grow at normal room temperatures (20–30°C). The count of mesophilic bacteria in honey is used as a general indicator to evaluate the quality and safety of the honey. An increase in the count of mesophilic bacteria in honey indicates contamination and spoilage of honey. Additionally, the count of mesophilic bacteria in honey is used as an indicator to monitor the process of production and storage of honey (Krishnan et al., 2021).

## 5 CONCLUSION

Ultrasound treatment is a non‐thermal processing technology that has the potential to enhance the quality and safety of honey. The present study has shown that ultrasound treatment can effectively reduce moisture content, increase pH, reduce acidity, reduce HMF levels, increase phenol content, and reduce the number of aerobic mesophilic bacteria. These findings suggest that ultrasound treatment can be used to control the physical, biochemical, antioxidant, and antimicrobial properties of honey, leading to improved overall quality. In addition to the benefits listed above, ultrasound treatment can also help reduce the undesirable effects of crystallization in honey. This is because ultrasound waves can break down the large sugar crystals that are responsible for crystallization. This makes ultrasound treatment a promising technology for the food industry. However, further investigation is needed to evaluate the optimal conditions for ultrasound treatment and to investigate the effects of ultrasound treatment on other types of honey. Additionally, it is important to evaluate the economic feasibility of ultrasound treatment for industrial honey production. Overall, the present study provides strong evidence that ultrasound treatment is a promising technology for improving the quality and safety of Iranian industrial honey.

## AUTHOR CONTRIBUTIONS

**Safa Rabbani:** Conceptualization (equal); data curation (equal). **Seyed Amir Ali Anvar:** Project administration (equal); visualization (equal). **Sara Allahyaribeik:** Investigation (equal); methodology (equal); writing – original draft (equal). **Behrooz Jannat:** Methodology (equal); resources (equal); writing – original draft (equal). **Hamed Ahari:** Investigation (equal); methodology (equal).

## CONFLICT OF INTEREST STATEMENT

The authors declare that they have no competing interests.

## Data Availability

All the data analyzed during this study are included in this published article.
